# The Efficacy of Mebeverine in the Treatment of Irritable Bowel Syndrome—A Systematic Review

**DOI:** 10.3390/jcm11041044

**Published:** 2022-02-17

**Authors:** Jaroslaw Daniluk, Ewa Malecka-Wojciesko, Barbara Skrzydlo-Radomanska, Grazyna Rydzewska

**Affiliations:** 1 Department of Gastroenterology and Internal Medicine, Medical University of Bialystok, 15-276 Bialystok, Poland; 2 Department of Digestive Tract Diseases, Medical University of Lodz, 90-153 Lodz, Poland; ewa.malecka-panas@umed.lodz.pl; 3 Department of Gastroenterology with Endoscopy Laboratory, Medical University of Lublin, 20-090 Lublin, Poland; barbara.skrzydlo-radomanska@umlub.pl; 4 Clinical Department of Internal Medicine and Gastroenterology with Inflammatory Bowel Disease Unit, CSK MSWiA, 02-507 Warsaw, Poland; grazyna.rydzewska@cskmswia.gov.pl; 5 Collegium Medicum, Jan Kochanowski University, 25-516 Kielce, Poland

**Keywords:** irritable bowel syndrome, mebeverine, systematic review

## Abstract

Background: Irritable bowel syndrome (IBS) is a common gastrointestinal tract disorder, affecting 10–20% of adults worldwide. Mebeverine is an antispasmodic agent indicated for the symptomatic treatment of abdominal pain caused by intestinal smooth muscle spasms and intestinal functional disorders in the course of IBS. The aim of this article was to perform a systematic literature review and update previous overviews of the efficacy and safety of mebeverine treatment in IBS. Methods: Major electronic medical databases, PubMed, EMBASE and Cochrane, were systematically searched from January 1965 to January 2021. Results: Twenty-two studies met our inclusion criteria, including 19 randomised trials, two observational retrospective studies, and one non-randomised, single-blinded study. Six studies reported a significant decrease in abdominal pain after mebeverine treatment (*p*-values ranging from <0.05 to <0.001). Only three studies showed no improvement after mebeverine treatment in terms of the severity of abdominal pain or discomfort. Some of the included studies also showed significant improvements in abnormal bowel habits, abdominal distension, as well as stool frequency and consistency. Adverse events were rare and associated mainly with IBS symptoms. Conclusions: Mebeverine is an effective treatment option in IBS, with a good safety profile and low frequency of adverse effects.

## 1. Introduction

Irritable bowel syndrome (IBS) is a common gastrointestinal tract disorder, affecting 10–20% of adults worldwide [1]. Almost half of all IBS patients report their first symptoms before age 35, which negatively affects their professional activity [1]. The main clinical manifestation of IBS is abdominal pain related to defecation, in addition to a change of bowel habit or stool consistency [1]. Although the pathogenesis of IBS is not fully understood, gut-brain axis disturbances, intestinal microbiota dysbiosis, abnormal gut motility, visceral hypersensitivity, and local immune system dysfunction are all thought to influence disease development [2].

Diagnosis of IBS is primarily based on clinical symptoms, and additional tests are not routinely recommended. While several diagnostic criteria have been developed over the years (e.g., Manning, Kruis, Rome I–IV), there is still no gold standard for diagnosing IBS [3,4]. Currently, the Rome IV criteria are recommended to establish the diagnosis of the disease [5]. Based on these criteria, IBS with predominant constipation (IBS-C) can be distinguished from IBS with predominant diarrhoea (IBS-D) and IBS with mixed bowel habits (IBS-M), or IBS may be unclassified (IBS-U) [2].

Due to the chronic nature of the disease, treatment remains challenging and depends on the symptoms. Therapeutic options consist of non-pharmacological management like lifestyle and dietary modifications and pharmacological treatment, depending on the predominant symptoms, i.e., abdominal pain, constipation, or diarrhoea [6]. Antispasmodics are currently the recommended treatment of choice for IBS patients with abdominal pain, as previous meta-analyses have reported some advantages over placebo [7]. However, the effectiveness of different antispasmodic agents varies.

Mebeverine is an antispasmodic agent indicated for the symptomatic treatment of abdominal pain caused by intestinal smooth muscle spasms and intestinal functional disorders in the course of IBS. It acts by relaxing the intestinal muscles and regulating bowel function. Studies assessing the effectiveness of mebeverine in IBS date back to the 1960s, even before the Rome I criteria for diagnosing IBS were released in 1992 [8]. The last systematic review and meta-analysis of mebeverine efficacy in IBS was performed more than ten years ago [9]. Therefore, this study aimed to systematically review currently available data to assess the effectiveness and safety of mebeverine in patients who were diagnosed according to IBS diagnostic criteria (i.e., Rome I–IV and other than Rome) and who suffer from bowel symptoms, including abdominal pain and discomfort, abdominal distension, abnormal bowel habits, bloating, constipation, and diarrhoea.

## 2. Materials and Methods

### 2.1. Information Sources and Searches

The systematic literature review was performed according to the previously developed, detailed protocol and the Preferred Reporting Items for Systematic Reviews and Meta-Analyses (PRISMA) guidelines [10]. Major electronic medical databases, PubMed, EMBASE and Cochrane, were systematically searched from January 1965 to January 2021 to estimate the treatment effects of mebeverine in patients with IBS. The keywords used for the search were: mebeverine, mebeverin, duspatalin, spasmotalin, and 4-(ethyl-(4-methoxy-alpha-methylphenethyl)aminobutyl) veratrate (Tables S1–S3).

### 2.2. Study Selection and Quality Evaluation

Titles and abstracts of all obtained articles were assessed for inclusion in the study. We used the PICO framework to develop our search strategy’s inclusion and exclusion criteria (Table 1). Inclusion criteria were: patients with a diagnosis of IBS; mebeverine treatment (regardless of dose and duration); specific outcomes (abdominal pain or discomfort, abdominal distension, abnormal bowel habits, bloating, constipation, diarrhoea, stool frequency and consistency, nausea, anxiety and depression); studies (experimental or observational) including ≥ten patients; and studies published in English. Studies involving mebeverine treatment in combination with another drug, cognitive therapy or diet, as well as case studies or secondary studies (i.e., systematic reviews, reviews), were excluded from the analysis.

Two authors independently (J.D., B.S.-R) reviewed each title, abstract, and full-text to evaluate study quality and eligibility according to the abovementioned inclusion/exclusion criteria. The same reviewers independently extracted data for measured outcomes. All discrepancies between the reviewers were resolved through discussion.

The risk of bias in experimental studies was assessed by the revised Cochrane risk-of-bias tool for randomized trials (RoB 2) [11]. The quality of observational studies was evaluated using the NICE checklist [12]. The Cochrane tool is based on five distinct domains for assessing potential sources of bias: (1) randomization process, (2) deviations from intended interventions, (3) missing outcome data, (4) measurement of the outcome, (5) selection of the reported results. For each domain, the risk of bias is judged as “low”, “high” or “some concerns”. Because of its comprehensiveness, the RoB2 tool became the standard approach to assess the risk of bias for randomized trials. The NICE checklist contains valid questions about the reliability of the methodology of observational studies, including the clarity of the study purpose and selection criteria, consecutiveness, and the direction of observation. The result is presented on 8-point scale, where the higher score indicates better quality of the study.

### 2.3. Data Items and Extraction

Data on the criteria used for the diagnosis of IBS, the characteristics of the treated groups, the treatment length and dosages were extracted for each of the studies based on the previously prepared form. Quantitative data on the severity or frequency of the following symptoms (i.e., measured outcomes) were also obtained: abdominal pain or discomfort, abdominal distension, abnormal bowel habits, bloating, constipation, diarrhoea, stool frequency and consistency, nausea, anxiety and depression. The *p*-value for each specific outcome was also extracted when available.

### 2.4. Statistical Analysis

Given the qualitative and narrative nature of this systematic review, no statistical analyses were performed.

## 3. Results

### 3.1. Overview of Included Studies

**Figure 1:**
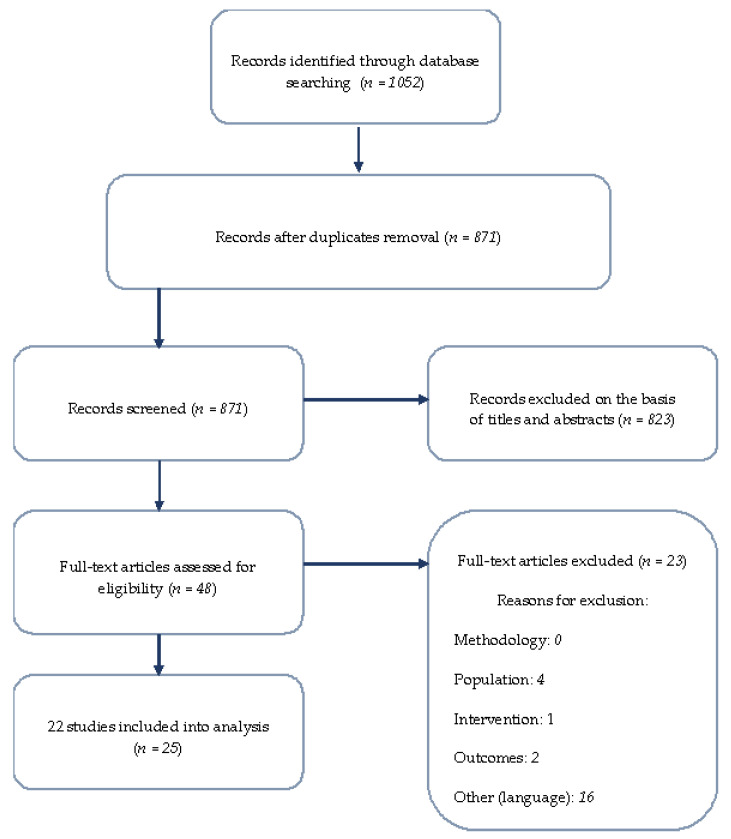
Flow chart of literature search strategy according to PRISMA.

The search strategy yielded 871 unique papers (after duplicate removal), with 52 publications warranting further assessment based on their titles and abstracts (Figure 1). Of these, 25 publications (22 studies) met our inclusion criteria (listed in Table 2) [13,14,15,16,17,18,19,20,21,22,23,24,25,26,27,28,29,30,31,32,33,34,35,36,37,38].

Among the 22 studies included in our analysis, one used the Manning criteria, two used the Kruis criteria, two used the Rome I criteria, two used the Rome II criteria, five used the Rome III criteria, three used the Rome IV criteria, and seven used different or unspecified criteria for diagnosing IBS.

Of the 22 studies, 19 were randomised trials; two were observational studies [13,14], and one was a non-randomised, single-blinded study [15]. The number of patients included in the studies ranged from 20 to 464 patients, and data about percentages of patients with specific IBS subtypes were present in 12 studies. Mebeverine was compared with placebo (seven studies) [15,16,17,18,19,20,21], trimebutine (two studies) [22,23], octilonium bromide (two studies) [20,24], pinaverium bromide (two studies) [14,25], alosetron (one study) [26], herbal combination (one study) [27], methylcellulose (one study) [16], ramosteron (one study) [28], probiotic (one study) [13], cumin sofouf (one study) [29], Luvos^®^ Healing Earth (one study) [30] and alverine citrate (one study) [31]. Only three studies compared different doses of mebeverine [32,33,34]. The treatment period varied from 2 to 16 weeks across the included studies. The most frequently evaluated symptoms were abdominal pain, bloating, and stool frequency and consistency (Table 2).

Only one of the included in the systematic review trials was at low risk of bias (Table S4) [21]. Some concerns regarding the risk of bias were present in eleven studies [16,20,22,23,24,25,26,28,32,33,34]. These concerns usually resulted from a lack of the description of the randomization process and doubts regarding the concealment of the allocation sequence. Another frequent issue was the high probability of using an inappropriate type of analysis to estimate the effect of assignment to intervention (‘as treated’ or ‘as completed’). The remaining eight studies were judged as being at a high risk of bias [15,17,18,19,27,29,30,31]. The main identified risks for these studies included the possible high impact of missing outcomes on results and the selection of the reported results. The quality of the observational studies was variable (Table S5). An article published by Hou et al. [14] was judged as having a good quality, while Guslandi [13] reported his results only as abstract, so the assessment of the quality of the study may be underestimated.

### 3.2. Efficacy of Mebeverine

Intestinal symptoms associated with IBS were assessed in all 22 studies, although only two studies [16,29] used the IBS Symptom Severity Scale (IBS-SSS) to measure abdominal pain, its duration, abdominal distension/tightness, bowel habits, and quality of life (QOL). The efficacy of mebeverine on six major intestinal symptoms are detailed below.

#### 3.2.1. Abdominal Pain and Discomfort

Nineteen studies evaluated the effect of mebeverine on the severity, frequency, and intensity of abdominal pain and discomfort in IBS patients, with a total of 1824 patients analysed in the mebeverine arm. A positive effect is presented as a percentage of patients with decreased symptoms or improved abdominal pain score compared to baseline. The treatment period varied from 2 to 12 weeks.

As shown in Table S6, six studies detected a significant decrease in abdominal pain score after mebeverine treatment compared to baseline (*p*-values ranging from <0.05 to <0.001) [21,23,28,29,30,31]. Eleven more studies showed mebeverine had a beneficial effect on reducing abdominal pain and discomfort, although the authors did not specify the statistical significance of the observed change [13,14,15,18,20,22,26,27,32,33,34]. Additionally, two trials proved the superiority of mebeverine over placebo in terms of abdominal pain reduction [18,20]. In two of nineteen studies, the beneficial effect of mebeverine was uncertain or insignificant [19,25]. Kruis et al. showed that initial abdominal pain improved only in 23% of patients treated with mebeverine; however, the compliance of treatment was below 50% [19]. Lu et al. reported that a similar percentage of patients suffered from abdominal pain before and after mebeverine treatment. However, the lack of significance possibly results from mild baseline pain intensity in about two-thirds of patients [25].

Three articles assessed the frequency of abdominal pain or discomfort after mebeverine treatment. All those studies found that mebeverine treatment reduced numerically abdominal pain frequency compared to baseline [22,27,29]. The improvement from baseline was statistically significant only in one trial [29], as no statistical calculations in the remaining two studies were performed [22,27].

#### 3.2.2. Abdominal Distension

The effect of mebeverine on abdominal distension was assessed in three studies, totalling 109 patients (Table S7) [18,27,32]. All three studies showed that mebeverine had a positive effect on abdominal distension. In particular, Prout et al. found the severity of the distension score was significantly lower in the mebeverine group than the placebo group after eight weeks of treatment (1.692 vs. 1.839; *p* < 0.05) [18]. The percentage of IBS patients with abdominal distension after mebeverine treatment compared to baseline was also numerically reduced in the two other studies [27,32].

#### 3.2.3. Abnormal Bowel Habits and Bloating

The effect of mebeverine on abnormal bowel habits in IBS patients was evaluated in five studies [15,18,19,28,31], as well as bloating [13,24,25,30,33], totalling 381 patients (Table S8). Four studies showed that mebeverine treatment numerically reduced abnormal bowel habits [15,18,28,31], while benefits in one study were found uncertain [19]. The authors of the two studies performed statistical calculations: Lee et al. reported a significant reduction in the number of abnormal bowel habits among patients taking mebeverine compared to baseline (*p* < 0.001) [28], and Prout et al. showed a significant reduction in pain during bowel movements in mebeverine-treated groups (i.e., 1.188 and 1.248 for the mebeverine low and high dose, respectively) compared to placebo (1.374; *p* < 0.05); however, the clinical value of the change was unclear for the authors [18]. Bloating was also reduced in all five studies [13,24,25,30,33]; however, only in one study, the statistics were calculated, i.e., Chang et al. reported that abdominal bloating assessed on a visual analogue scale (VAS) reduced from 4.7 (6.6) at baseline to 1.3 (4.6) at week eight (*p* < 0.001) [24].

#### 3.2.4. Constipation and Diarrhoea

The effect of mebeverine on constipation and diarrhoea was analysed in three studies for both constipation [20,32,33] and diarrhoea [13,20,30], with all studies showing a reduction of these symptoms (Table S9). Specifically, treatment with mebeverine for 3 to 6 weeks caused the resolution of constipation in 62% to 79% of patients [32,33]. Similarly, six weeks of mebeverine treatment caused diarrhoea to improve or disappear [13]. Finally, one study showed that the severity of both constipation and diarrhoea were significantly lower after mebeverine treatment compared to placebo (*p* < 0.001) [20].

#### 3.2.5. Stool Frequency and Consistency

Eight studies (totalling 722 patients) evaluated the effect of mebeverine on stool frequency [21,23,24,25,26,27,28,30], with a treatment period ranging from 2 to 12 weeks (Table S10). All eight studies showed a reduction in stool frequency after mebeverine therapy, with five reporting a statistically significant change [21,23,24,25,28]. In one study, the change from baseline was insignificant [30], while in remaining two studies, no statistical calculations were made [26,27]. Five studies, including 608 patients, determined the influence of mebeverine on stool consistency [23,25,26,27,28]. All five studies showed mebeverine had a favourable effect on stool consistency, with three studies [23,25,28] revealing a statistically significant improvement.

#### 3.2.6. Nausea, Anxiety and Depression

The effect of mebeverine on nausea was only evaluated in one study (Table S11), in which the severity of nausea score was significantly lower in the mebeverine group (1.170) than in the placebo (1.311; *p* < 0.05) [18]. The effect of mebeverine on depression and anxiety, two very common symptoms in IBS patients, was evaluated in the MIBS (Management of IBS in Primary Care) trial (Table S9) [35]. The mean HADS (Hospital Anxiety and Depression Scale) score for anxiety was reduced from 9.23 at baseline to 8.7 at week six and to 8.2 at week 12 in patients treated with mebeverine. Similarly, 85% and 78% of patients reported normal HADS scores for depression after 6 and 12 weeks of treatment, respectively [35]. Furthermore, Prout et al. reported that the severity of anxiety score was significantly lower in the mebeverine high dose group (1.578) than the placebo group (1.704; *p* < 0.05), nevertheless, the authors were not able to determine if this difference was clinically significant [18].

### 3.3. Safety Assessment

Nineteen studies examined the prevalence and severity of adverse events after mebeverine treatment (Table S12). Generally, adverse events were rare and associated mainly with IBS symptoms. According to the authors’ opinion, serious adverse events were reported in three studies [24,26,34], although they were at a low prevalence (ranging from 1.8% to 8.6%) and, according to the authors’ opinion, were unlikely to be related to mebeverine.

## 4. Discussion

Despite its high prevalence, effective treatment for IBS remains challenging. Current guidelines recommend dietary and lifestyle modifications, as well as pharmacological therapies [4,39,40]. The treatment strategy should be based on the most predominant symptoms, patient preferences and expectations. Since IBS is a chronic condition with periods of exacerbation and remission of symptoms, treatment is long-lasting, and therapy outcomes vary between individuals.

Our systematic literature review results demonstrate that mebeverine is an effective and safe therapeutic option in patients with IBS. In the majority of patients included in trials, mebeverine therapy was associated with the reduction of diverse intestinal symptoms, including abdominal pain and discomfort, abdominal distension, abnormal or irregular bowel habits, bloating, and disturbances in stool frequency and consistency.

Mebeverine is an antispasmodic agent that works directly on intestinal smooth muscles and may also have a local anaesthetic effect and weak atropine-like activity. Current guidelines recommend antispasmodics as the drug of choice for IBS patients with a pain predominance [39,40,41]. Indeed, a systematic review of 26 randomised clinical trials (RCTs) including 2811 patients with IBS and 13 different antispasmodics showed significant improvement of IBS symptoms upon antispasmodic treatment compared to placebo (risk ratio [RR] of IBS symptoms not improving 0.65; 95% confidence interval [CI], 0.56–0.76; *p* < 0.00001; number needed to treat [NNT] 5; 95% CI, 4–8) [39]. However, antispasmodics are a heterogeneous group of drugs with different mechanisms of action. Currently, hyoscine and drotaverine are recommended for IBS treatment over the other antispasmodic agents [39,40]. Other drugs with confirmed efficacy in reducing IBS symptoms are otilonium, pinaverium, cimetropium, and dicyclomine.

A previous literature review indicated that mebeverine has no statistically significant beneficial effect on IBS symptoms (RR 1.18; 95% CI, 0.93–1.50) [39,40]. However, according to the authors, most trials included in the analysis were of poor quality, with a small sample size and significant heterogeneity in the results [40]. Indeed, only six studies totalling 351 patients treated with mebeverine were included in this previous systematic review. The latest meta-analysis published in 2010 also did not show any clinical improvement or reduction of abdominal pain in IBS patients after mebeverine treatment. These results are in contrast to our findings, which showed a beneficial effect of mebeverine on IBS symptoms. This difference may be caused, at least partially, by the inclusion of new studies in our review compared to the previous analysis (which was published almost ten years ago and only included six studies involving 279 patients treated with mebeverine). In addition, abdominal pain was a prevalent symptom in only one of these studies.

Our study added ten recently published articles (eight randomised and two retrospective observational studies), totalling 1945 patients treated with mebeverine. Abdominal pain and discomfort were evaluated in nineteen studies, including 1824 patients in the mebeverine arm. Six studies detected a significant decrease in the abdominal pain score from baseline, and eleven more studies showed a numerical improvement in the reduction of abdominal pain and discomfort after mebeverine (although statistical calculations were not available). Moreover, three studies showed a reduction of the pain frequency from baseline. Our results are similar to those reported by Poynard et al. [42], in which a meta-analysis of showed a significant reduction of pain upon smooth muscle relaxants including mebeverine compared to placebo (odds ratio 1.65; 95% Cl: 1.26–2.17).

Although pain is the major clinical manifestation of IBS, other symptoms are also common due to the multifaceted nature of the disease. Indeed, we showed that mebeverine also had a positive effect on other IBS symptoms, including improvements in abdominal distension, stool frequency, and abnormal bowel habits. These results are in accordance with a previous meta-analysis, which confirmed the beneficial effect of myorelaxants on abdominal distension (OR 1.46: 95% CI: 1.10–1.94, *p* = 0.008) and a significant global improvement (OR 2.04; 95% Cl: 1.15–3.63); however, antispasmodics had no effect on constipation or bowel transit time [42].

Our analysis included two studies comparing mebeverine with serotonin (5-HT3) receptor antagonists (ramosetron and alosetron), which are new drugs developed for patients with IBS without constipation [26,28,38]. Lee et al. showed mebeverine had comparable effectiveness to ramosetron in male patients with IBS-D [28,38]. Specifically, there were no differences in the global IBS symptoms, abdominal pain/discomfort, abnormal bowel habits, responder rates (37% vs. 38% on the intention-to-treat [ITT] analysis), or safety profiles between the two drugs [28,38]. Conversely, Jones et al. found alosetron was superior to mebeverine in terms of pain relief and improvement of bowel function in non-constipated females with IBS [26]. However, as alosetron is currently unavailable in many countries, mebeverine could still be useful, particularly in treating males and constipated females with IBS.

IBS has a substantial impact on health-related quality of life (QOL): the QOL of IBS patients is lower than that of diabetic patients, individuals suffering from end-stage kidney disease or those with gastroesophageal reflux disease [43]. An observational, prospective study of patients with IBS across four countries (Poland, Egypt, Mexico, and China) showed a significant improvement in IBS-related QOL after eight weeks of mebeverine treatment [14]. The observational study showed that mebeverine had beneficial effects on gastrointestinal symptoms, with a significant decrease in the severity of abdominal pain and discomfort and improvements in stool frequency and consistency, bloating, abdominal distension, and urgency [14].

Our systematic review also showed that mebeverine is a safe drug, with few adverse effects compared to placebo. These results are consistent with previous reports on the safety of this drug [9,44]. For example, the meta-analysis by Poynard et al. found 98% of IBS patients treated with mebeverine have no adverse effects (compared to 99% of patients in the placebo group) [42].

**Figure 2:**
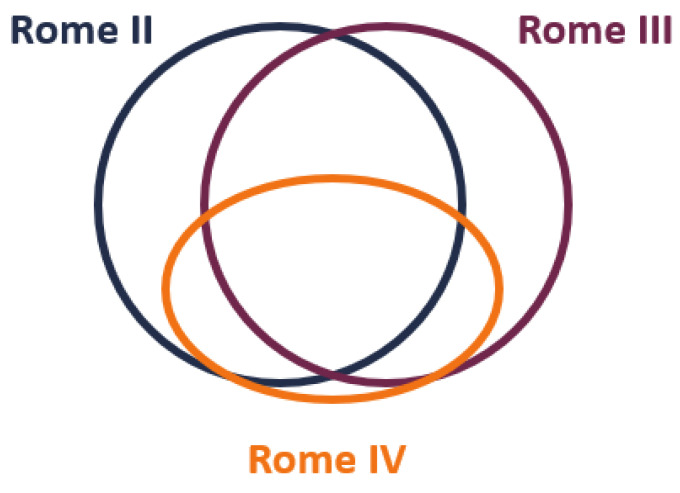
Venn diagram for range and relationship between IBS diagnostic criteria Rome II, III and IV.

Our study has some limitations. First, current guidelines recommend the use of the Rome IV criteria for the diagnosis of IBS [5], and only three studies in our analysis used Rome IV criteria, while the others were performed on patients with bowel disorders who met older IBS diagnostic criteria (i.e., Rome I–III and other than Rome). Studies comparing the diagnostic IBS criteria (Manning, Rome I, Rome II, Rome III, and Rome IV) suggest Rome IV has a narrower IBS definition (Table S13 and Table S14); therefore, the Rome IV IBS population likely reflects a subgroup of Rome II and III IBS patients with more severe gastrointestinal symptomatology, psychological comorbidities, and lower QOL (Figure 2) [45,46,47,48,49,50,51]. However, there is a large group of patients with less severe intestinal symptoms who, despite fulfilling older Rome criteria, are not considered IBS patients. Hence, the therapeutic effect of mebeverine effect may be different for groups meeting varying diagnostic criteria.

The second limitation is the difficulty in analysing heterogeneous studies with different patient characteristics, treatment durations, and endpoints. Fortunately, most studies included in our analysis were randomised trials with well-characterised outcomes. Our analysis is also limited by the inability to present results for each IBS subtype due to limited data. This particular concerns patients with IBS-C who were a minority in most of the included studies [16,23,24,26,29], so generalization of mebeverine benefits on this population is burdened with uncertainty.

Another limitation of this analysis is the lack of the direct comparison of mebeverine efficacy with placebo, which might be challenging, due to the substantial placebo effect and spontaneous improvement of symptoms in IBS. Pitz et al. showed in the previous meta-analysis that response on placebo in terms of abdominal pain ranges from 24% to 70%, with the mean value of 27.5% [52]. In the studies included in our systematic review, abdominal pain improvement during mebeverine treatment ranged from 23% to 96%, with an average value estimated at 53%, which means that the efficacy of mebeverine is about 25 percentage points higher in comparison with placebo. Furthermore, although we did not perform a formal meta-analysis, our literature search was detailed and followed PRISMA guidelines, and our final analysis included a large number of patients treated with mebeverine.

Despite our results showing that mebeverine may be considered an effective and safe treatment option for patients with IBS, the applicability of our study is limited. Mebeverine is not available in many countries, including the USA.

## 5. Conclusions

In conclusion, mebeverine is an effective treatment for a wide range of IBS patients who are suffering from abdominal pain and discomfort, distension, abnormal or irregular bowel habits, bloating, constipation and diarrhoea, but who do not necessarily fulfil the recent IBS criteria (Rome IV). We found that mebeverine has a good safety profile, with a low frequency of adverse effects.

## Figures and Tables

**Table 1:** Inclusion and exclusion criteria for systematic literature search.

PICOS	Inclusion Criteria	Exclusion Criteria
Population	Patients with a diagnosis of IBS	Functional gastrointestinal disorders other than IBS
Intervention	Mebeverine, regardless of the dose and duration of treatment	Mebeverine in combination with another drug, cognitive therapy or diet
Comparators	No restrictions	x
Outcomes	Severity or frequency of bowel symptoms: abdominal pain or discomfort, abdominal distension, abnormal bowel habits, bloating, constipation, diarrhoea and other	x
Study (methodology)	Studies (experimental or observational) including ≥10 patients	Case studies, secondary studies (systematic reviews, reviews)

**Table 2:** Characteristics of the included studies.

Study Name	Population	Diagnostic Criteria	Mebeverine Arm			Evaluated Symptoms
			N	Dose	Treatment Period	
Lu 2000 [25]	IBS-D: 100%	Manning criteria	46	100 mg 3 times daily	2 weeks	Abdominal pain, bloating, stool frequency, stool consistency, incomplete evacuation, stool with mucus
Van Outryve 1995 [33]	IBS NOS: 100%	Kruis criteria	60	135 mg, 2 tablets 3 times daily or sustained release 200 mg, 2 tablets twice daily (crossing-over)	6 weeks	Abdominal pain, bloating, flatulence, constipation
Schaffstein 1990 [22]	IBS-C100% ^a^	Kruis criteria	99	135 mg 3 times daily	4 weeks	Abdominal pain
Jones 1999 [26]	IBS-D: 71%, IBS-C: 5%, IBS-M: 24%	Rome I	304	135 mg 3 times daily	12 weeks	Pain and discomfort, urgency, stool frequency, stool consistency
Gilbody 2000 [34]	IBS NOS: 100%	Rome I	184	135 mg 3 times daily or 200 mg twice daily	8 weeks	Abdominal pain
Chang 2011 [24]	IBS-D: 72%, IBS-C: 21%, IBS-M: 7%	Rome II	58	100 mg 3 times daily	8 weeks	Bloating, flatulence, stool frequency
Rahman 2014 [23]	IBS-D: 67%, IBS-C: 33% ^b^	Rome II	70	135 mg twice daily	6 weeks	Abdominal pain, flatulence, stool frequency, stool consistency
Sahib 2013 [27]	IBS NOS: 100%	Rome III	20	135 mg 3 times daily	8 weeks	Pain, abdominal distension, urgency, stool frequency, stool consistency, incomplete evacuation, the passing of mucus
MIBS trial 2013 [16,35,36,37]	IBS-D: 30%, IBS-C: 11%, IBS-M: 57%	Rome III	43	135 mg 3 times daily	6 weeks	IBS-SSS (severity of abdominal pain, duration of abdominal pain, abdominal distension/tightness, bowel habit, quality of life), anxiety, depression
Lee 2011 [28,38]	IBS-D: 100%	Rome III	168	135 mg 3 times daily	4 weeks	Abdominal pain/discomfort, abnormal bowel habits, urgency, stool frequency, stool consistency
Guslandi 2011 [13]	IBS-D: 100% ^c^	Rome III	28	200 mg twice daily	6 weeks	Abdominal discomfort, bloating, diarrhoea
Hou 2014 [14]	IBS-D: 33%, IBS-C: 33%, IBS-M: 33%	Rome III	464	135 mg 3 times daily or 200 mg twice daily or prolonged-release 200 mg twice daily	8 weeks	Abdominal pain/discomfort, quality of life
Chakraborty 2019 [21]	IBS -D: 100%	Rome IV	20	200 mg twice daily controlled release	8 weeks	Abdominal pain, stool frequency, quality of life
Hatami 2020 [29]	IBS-D: 22.5%, IBS-C: 12.5%, IBS-M: 65%	Rome IV	40	200 mg twice daily sustain release	4 weeks	Abdominal pain, flatulence, quality of life
Mokhtare 2018 [30]	IBS-D: 100%	Rome IV	36	135 mg twice daily	4 weeks	Abdominal pain, bloating, diarrhoea, stool frequency
Connell 1965 [17]	IBS NOS: 100%	NR	20	100 mg 4 times daily	12 weeks	Abdominal cramps, disturbance of bowel habit
Baume 1972 [15]	IBS NOS: 100%	Truelove and Reynell diagnostic criteria for IBS	59	50 mg, 2 tablets twice daily	2 weeks	Pain, abnormal bowel habits
Prout 1983 [18]	IBS NOS: 100%	NR	41	405 mg or 810 mg (crossing-over)	8 weeks	Abdominal pain, abdominal distension, pain on moving bowels, wind, nausea, anxiety
Kruis 1986 [19]	IBS-D: 18%, IBS-C: 36%, IBS-M: 46% ^d^	Own criteria	40	100 mg 4 times daily	16 weeks	Abdominal pain, irregular bowel habits, flatulence
Inauen 1994 [32]	IBS NOS: 100%	NR	48	135 mg 3 times daily or slow-release 200 mg twice daily	3 weeks	Abdominal pain, abdominal distension, constipation
Tudor 1986 [31]	IBS NOS: 100%	NR	37	135 mg	4 weeks	Abdominal pain, bowel habits
Capurso 1984 [20]	IBS NOS: 100%	NR	60	135 mg 3 times daily	2 weeks	Pain, flatulence, constipation or diarrhoea

IBS—irritable bowel syndrome; IBS-C: IBS with predominant constipation; IBS-D: IBS with predominant diarrhoea; IBS-M: IBS mixed type; N—number of patients; NOS—not otherwise specified; NR—not reported. ^a^—The population was described as patients without diarrhoea. ^b^—Data for 60 patients. ^c^—The population was described as patients without constipation. ^d^—Data for 120 patients, including placebo and bran subgroups.

## Data Availability

Not applicable.

## Supplementary Materials

The following supporting information can be downloaded at: https://www.mdpi.com/article/10.3390/jcm11041044/s1, Table S1: Search strategy for MEDLINE (via Pubmed), Table S2: Search strategy for Embase, Table S3: Search strategy for Cochrane, Table S4: Risk of bias (RoB 2) in experimental studies, Table S5: Quality assessment for observational studies (the NICE checklist), Table S6: Summary of the results of mebeverine studies with respect to the symptoms associated with abdominal pain and discomfort, Table S7: Summary of the results of mebeverine studies with respect to the symptoms associated with abdominal distension, Table S8: Summary of the results of mebeverine studies with respect to the symptoms associated with abnormal bowel habits and bloating, Table S9: Summary of the results of mebeverine studies with respect to the symptoms associated with constipation and diarrhoea, Table S10: Summary of the results of mebeverine studies with respect to the symptoms associated with stool frequency and consistency, Table S11: Summary of the results of mebeverine studies with respect to the symptoms associated with nausea, anxiety and depression, Table S12: Summary of the safety results, Table S13: Comparison of various IBS diagnostic criteria, Table S14: Prevalence of IBS in patients diagnosed by different criteria.

## References

[1] Canavan C., West J., Card T. (2014). The epidemiology of irritable bowel syndrome. Clin. Epidemiol..

[2] Drossman D.A. (2016). Functional Gastrointestinal Disorders: History, Pathophysiology, Clinical Features and Rome IV. Gastroenterology.

[3] Lacy B.E., Patel N.K. (2017). Rome Criteria and a Diagnostic Approach to Irritable Bowel Syndrome. J. Clin. Med..

[4] Moayyedi P., Mearin F., Azpiroz F., Andresen V., Barbara G., Corsetti M., Emmanuel A., Hungin A.P.S., Layer P., Stanghellini V. (2017). Irritable bowel syndrome diagnosis and management: A simplified algorithm for clinical practice. United Eur. Gastroenterol. J..

[5] Mearin F., Lacy B.E., Chang L., Chey W.D., Lembo A.J., Simren M., Spiller R. (2016). Bowel Disorders. Gastroenterology.

[6] Chey W.D., Kurlander J., Eswaran S. (2015). Irritable bowel syndrome: A clinical review. JAMA.

[7] Annaházi A., Róka R., Rosztóczy A., Wittmann T. (2014). Role of antispasmodics in the treatment of irritable bowel syndrome. World J. Gastroenterol..

[8] De Groote J., Standaert L. (1968). The effect of a new musculotropic subtance 9(Mebeverine) on irritable colon. Tijdschr. Gastroenterol..

[9] Darvish-Damavandi M., Nikfar S., Abdollahi M. (2010). A systematic review of efficacy and tolerability of mebeverine in irritable bowel syndrome. World J. Gastroenterol..

[10] Moher D., Liberati A., Tetzlaff J., Altman D.G., Group P. (2009). Preferred reporting items for systematic reviews and meta-analyses: The PRISMA statement. J. Clin. Epidemiol..

[11] Higgins J., Savovic J., Elbers R. (2019). Chapter 8: Assessing risk of bias in a randomized trial. Cochrane Handbook for Systematic Reviews of Interventions.

[12] NICE Quality of Case Series Form. http://www.nice.org.uk/guidance/cg3/resources/appendix-4-quality-of-case-series-form2.

[13] Guslandi M. (2011). Mebeverine plus saccharomyces boularii versus mebeverine alone in the treatment of irritable bowel syndrome without constipation: A retrospective analysis. Am. J. Gastroenterol..

[14] Hou X., Chen S., Zhang Y., Sha W., Yu X., El Sawah H., Afifi A.F., El-Khayat H.R., Nouh A., Hassan M.F. (2014). Quality of life in patients with Irritable Bowel Syndrome (IBS), assessed using the IBS-Quality of Life (IBS-QOL) measure after 4 and 8 weeks of treatment with mebeverine hydrochloride or pinaverium bromide: Results of an international prospective observational cohort study in Poland, Egypt, Mexico and China. Clin. Drug Investig..

[15] Baume P. (1972). Mebeverine, an effective agent in the irritable colon syndrome. Aust. N. Z. J. Med..

[16] Everitt H., Moss-Morris R., Sibelli A., Tapp L., Coleman N., Yardley L., Smith P.W.F., Little P. (2013). Management of irritable bowel syndrome in primary care: The results of an exploratory randomised controlled trial of mebeverine, methylcellulose, placebo and a self-management website. BMC Gastroenterol..

[17] Connell A.M. (1965). Physiological and clinical assessment of the effect of the musculotropic agent mebeverine on the human colon. Br. Med. J..

[18] Prout B.J. (1983). The treatment of irritable bowel syndrome. Two doses of mebeverine compared. Practitioner.

[19] Kruis W., Weinzierl M., Schüssler P., Holl J. (1986). Comparison of the therapeutic effect of wheat bran, mebeverine and placebo in patients with the irritable bowel syndrome. Digestion.

[20] Capurso L., Koch M., Tarquini M., Dezi A., Papi C., Fracasso P. (1984). The irritable bowel syndrome. A cross-over study of octilonium bromide, mebeverine and placebo. Clin. Trials J..

[21] Chakraborty D.S., Hazra A., Sil A., Pain S. (2019). Will controlled release mebeverine be able to surpass placebo in treatment of diarrhoea predominant irritable bowel syndrome?. J. Fam. Med. Prim. Care.

[22] Schaffstein W., Panijel M., Luettecke K. (1990). Comparative safety and efficacy of trimebutine versus mebeverine in the treatment of irritable bowel syndrome. A multicenter double-blind study. Curr. Res. Clin. Exp..

[23] Rahman M.Z., Ahmed D.S., Mahmuduzzaman M., Chowdhury M.S., Barua R., Ishaque S.M. (2014). Comparative efficacy and safety of trimebutine versus mebeverine in the treatment of irritable bowel syndrome. Mymensingh Med. J..

[24] Chang F.Y., Lu C.L., Luo J.C., Chen T.S., Chen M.J., Chang H.J. (2011). The evaluation of otilonium bromide treatment in Asian patients with irritable bowel syndrome. J. Neurogastroenterol. Motil..

[25] Lu C., Chen C., Chang F., Chang S., Kang L., Lu R., Lee S. (2000). Effect of a calcium channel blocker and antispasmodic in diarrhoea-predominant irritable bowel syndrome. J. Gastroenterol. Hepatol..

[26] Jones R.H., Holtmann G., Rodrigo L., Ehsanullah R.S., Crompton P.M., Jacques L.A., Mills J.G. (1999). Alosetron relieves pain and improves bowel function compared with mebeverine in female nonconstipated irritable bowel syndrome patients. Aliment. Pharm..

[27] Sahib A.S. (2013). Treatment of irritable bowel syndrome using a selected herbal combination of Iraqi folk medicines. J. Ethnopharmacol..

[28] Lee K.J., Kim N.Y., Kwon J.K., Huh K.C., Lee O.Y., Lee J.S., Choi S.C., Sohn C.I., Myung S.J., Park H. (2011). Efficacy of ramosetron in the treatment of male patients with irritable bowel syndrome with diarrhea: A multicenter, randomized clinical trial, compared with mebeverine. J. Neurogastroenterol. Motil..

[29] Hatami K., Kazemi-Motlagh A.H., Ajdarkosh H., Zargaran A., Karimi M., Shamshiri A.R., Ghadir M.R. (2020). Comparing the Efficacy of Cumin Sofouf With Mebeverine on Irritable Bowel Syndrome Severity and Quality of Life: A Double-blind Randomized Clinical Trial. Crescent. J. Med. Biol. Sci..

[30] Mokhtare M., Asadipanah M., Bahardoust M., Chaharmahali A., Sikaroudi M.K., Khoshdelnezamiha M., Davanloo F.A., Masoodi M., Bahadorizadeh L. (2018). Efficacy of adding Luvos^®^ Healing Earth supplementation to mebeverine in improving symptoms and quality of life of patients with diarrhea-predominant irritable bowel syndrome: A randomized clinical trial. Biomed. Res..

[31] Tudor G.J. (1986). A general practice study to compare alverine citrate with mebeverine hydrochloride in the treatment of irritable bowel syndrome. Br. J. Clin. Pract..

[32] Inauen W., Halter F. (1994). Clinical Efficacy, Safety and Tolerance of Mebeverine Slow Release (200 mg) vs Mebeverine Tablets in Patients with Irritable Bowel Syndrome. Drug Investig..

[33] Van Outryve M., Mayeur S., Meeus M.A., Rosillon D., Hendrickx B., Ceuppens M. (1995). A double-blind crossover comparison study of the safety and efficacy of mebeverine with mebeverine sustained release in the treatment of irritable bowel syndrome. J. Clin. Pharm..

[34] Gilbody J.S., Fletcher C.P., Hughes I.W., Kidman S.P. (2000). Comparison of two different formulations of mebeverine hydrochloride in irritable bowel syndrome. Int. J. Clin. Pract..

[35] Everitt H.A., Moss-Morris R.E., Sibelli A., Tapp L., Coleman N.S., Yardley L., Smith P.W., Little P.S. (2010). Management of irritable bowel syndrome in primary care: Feasibility randomised controlled trial of mebeverine, methylcellulose, placebo and a patient self-management cognitive behavioural therapy website. (MIBS trial). BMC Gastroenterol..

[36] Everitt H. Management of Irritable Bowel Syndrome in Primary Care (MIBS Trial). ClinicalTrials.gov, Internet. https://clinicaltrials.gov/ct2/show/NCT00934973.

[37] Clinicaltrialsregister.eu Internet Management of Irritable Bowel Syndrome in Primary Care: Feasibility Randomised Controlled Trial of Mebeverine, Methylcellulose, Placebo and a Patient Self-Management Cognitive Behavioural Therapy Website. (MIBS Trial). https://www.clinicaltrialsregister.eu/ctr-search/trial/2009-013426-16/GB.

[38] Lee K.J., Poong-Lyul R. (2011). A Randomized, Open Labeled, Multicenter Clinical Trial on the Effectiveness and Safety of the 5-HT3-Receptor Antagonist Ramosetron in Male Patients with Irritable Bowel Syndrome With Diarrhea: Comparison With Mebeverine. Gastroenterology.

[39] Moayyedi P., Andrews C.N., MacQueen G., Korownyk C., Marsiglio M., Graff L., Kvern B., Lazarescu A., Liu L., Paterson W.G. (2019). Canadian Association of Gastroenterology Clinical Practice Guideline for the Management of Irritable Bowel Syndrome (IBS). J. Can. Assoc. Gastroenterol..

[40] Pietrzak A., Skrzydło-Radomańska B., Mulak A., Lipiński M., Małecka-Panas E., Regula J., Rydzewska G. (2018). Guidelines on the management of irritable bowel syndrome: In memory of Professor Witold Bartnik. Prz. Gastroenterol..

[41] Jailwala J., Imperiale T.F., Kroenke K. (2000). Pharmacologic treatment of the irritable bowel syndrome: A systematic review of randomized, controlled trials. Ann. Intern. Med..

[42] Poynard T., Regimbeau C., Benhamou Y. (2001). Meta-analysis of smooth muscle relaxants in the treatment of irritable bowel syndrome. Aliment. Pharm..

[43] Gralnek I.M., Hays R.D., Kilbourne A., Naliboff B., Mayer E.A. (2000). The impact of irritable bowel syndrome on health-related quality of life. Gastroenterology.

[44] Poynard T., Naveau S., Mory B., Chaput J.C. (1994). Meta-analysis of smooth muscle relaxants in the treatment of irritable bowel syndrome. Aliment. Pharm..

[45] Vork L., Weerts Z.Z., Mujagic Z., Kruimel J.W., Hesselink M.A.M., Muris J., Keszthelyi D., Jonkers D.M.A.E., Masclee A.A.M. (2018). Rome III vs Rome IV criteria for irritable bowel syndrome: A comparison of clinical characteristics in a large cohort study. J. Neurogastroenterol. Motil..

[46] Ghoshal U.C., Abraham P., Bhatia S.J., Misra S.P., Choudhuri G., Biswas K.D., Chakravartty K., Dadhich S., Goswami B.D., Jayanthi V. (2013). Comparison of Manning, Rome I, II, and III, and Asian diagnostic criteria: Report of the Multicentric Indian Irritable Bowel Syndrome (MIIBS) study. Indian J. Gastroenterol..

[47] Sperber A.D., Shvartzman P., Friger M., Fich A. (2007). A comparative reappraisal of the Rome II and Rome III diagnostic criteria: Are we getting closer to the ‘true’ prevalence of irritable bowel syndrome?. Eur J. Gastroenterol. Hepatol..

[48] Park D.W., Lee O.Y., Shim S.G., Jun D.W., Lee K.N., Kim H.Y., Lee H.L., Yoon B.C., Choi H.S. (2010). The Differences in Prevalence and Sociodemographic Characteristics of Irritable Bowel Syndrome According to Rome II and Rome III. J. Neurogastroenterol. Motil..

[49] Bai T., Xia J., Jiang Y., Cao H., Zhao Y., Zhang L., Wang H., Song J., Hou X. (2017). Comparison of the Rome IV and Rome III criteria for IBS diagnosis: A cross-sectional survey. J. Gastroenterol. Hepatol..

[50] Palsson O.S., Whitehead W., Törnblom H., Sperber A.D., Simren M. (2020). Prevalence of Rome IV Functional Bowel Disorders Among Adults in the United States, Canada, and the United Kingdom. Gastroenterology.

[51] Patcharatrakul T., Thanapirom K., Gonlachanvit S. (2017). Application of Rome III vs. Rome IV Diagnostic Criteria for Irritable Bowel Syndrome (IBS) in Clinical Practice: Is the Newer the Better?. Gastroenterology.

[52] Pitz N., Cheang M., Bernstein C. (2005). Defining the predictors of the placebo response in irritable bowel syndrome. Clin. Gastrol. Hepatol..

