# Supplementary material for: The Efficacy of Mebeverine in the Treatment of Irritable Bowel Syndrome—A Systematic Review
Source: J Clin Med. 2022 Feb 17;11(4):1044. doi: 10.3390/jcm11041044 (PMC8879004; doi:10.3390/jcm11041044)
Supplement: Supplementary file 1 [file jcm-11-01044-s001.zip › jcm-1586401-supplementary.pdf]

Supplementary Materials

Table S1. Search strategy for MEDLINE (via PubMed).

#	Term	Score
#1	mebeverine	184
#2	mebeverin*	189
#3	"mebeverine" [Supplementary Concept]	127
#4	Duspatalin	187
#5	Spasmotalin	184
#6	"4-(ethyl-(4-methoxy-alpha-methylphenethyl)aminobutyl) veratrate"	0
#7	#1 OR #2 OR #3 OR #4 OR #5 OR #6	191

Date of search: 4 January 2021.

Table S2. Search strategy for Embase.

#	Term	Score
#1	mebeverine AND [embase]/lim	769
#2	mebeverin* AND [embase]/lim	775
#3	‘mebeverine’/exp AND [embase]/lim	763
#4	duspatalin AND [embase]/lim	85
#5	spasmotalin AND [embase]/lim	0
#6	‘4-(ethyl-(4-methoxy-alpha-methylphenethyl)aminobutyl) veratrate’ AND [embase]/lim	0
#7	#1 OR #2 OR #3 OR #4 OR #5 OR #6	775

Date of search: 4 January 2021.

Table S3. Search strategy for Cochrane.

#	Term	Score
#1	mebeverine AND	84
#2	mebeverin*	86
#3	duspatalin	0
#4	spasmotalin	0
#5	‘4-(ethyl-(4-methoxy-alpha-methylphenethyl)aminobutyl) veratrate’	0
#6	#1 OR #2 OR #3 OR #4 OR #5	86

There was no MeSH term for "mebeverine". Date of search: 4 January 2021.

Table S4. Risk of bias (RoB 2) in experimental studies.

Study name	Risk of bias					
	**Domain 1 (randomization process)**	**Domain 2 (deviations from intended interventions)**	**Domain 3 (missing outcome data)**	**Domain 4 (measurement of the outcome)**	**Domain 5 (selection of the reported result)**	**Overall risk of bias**
Lu 2000	Some concerns	Some concerns	Low	Low	Some concerns	Some concerns
Van Outryve 1995	Some concerns	Some concerns	Low	Low	Low	Some concerns
Schaffstein 1990	Some concerns	Some concerns	Low	Low	Low	Some concerns
Jones 1999	Some concerns	Low	Low	Low	Low	Some concerns
Gilbody 2000	Some concerns	Low	Low	Low	Low	Some concerns
Chang 2011	Low	Low	Some concerns	Low	Low	Some concerns
Rahman 2014	Some concerns	Some concerns	Some concerns	Some concerns	Some concerns	Some concerns
Sahib 2013	Some concerns	Some concerns	High	Some concerns	High	High
MIBS trial 2013	Some concerns	Low	Low	Low	Low	Some concerns
Lee 2011	Some concerns	Some concerns	Low	Some concerns	Some concerns	Some concerns
Chakraborty 2019	Low	Low	Low	Low	Low	Low
Hatami 2020	Some concerns	High	High	Low	Low	High
Mokhtare 2018	Some concerns	Some concerns	Some concerns	Some concerns	High	High
Connell 1965	Some concerns	Some concerns	Some concerns	High	High	High
Baume 1972	Some concerns	Some concerns	Low	High	Low	High
Prout 1983	Some concerns	Some concerns	Some concerns	Low	High	High
Kruis 1986	Some concerns	Some concerns	High	Low	Low	High
Inauen 1994	Some concerns	Some concerns	Low	Low	Low	Some concerns
Tudor 1986	Some concerns	Some concerns	Some concerns	Low	High	High
Capurso 1984	Low	Some concerns	Some concerns	Low	Low	Some concerns

Table S5. Quality assessment for observational studies (the NICE checklist).

Study name	Quality assessment for observational studies								
	Multi-centre study	Clearly described aim	Qualification criteria	Clear outcomes definition	Prospective data	Consecutive recruitment	Clear findings description	Outcomes stratification	Overall quality
Guslandi 2011	0	1	0	1	0	0	1	0	3/8
Hou 2014	1	1	1	1	1	0	1	1	7/8

Table S6. Summary of the results of mebeverine studies with respect to the symptoms associated with abdominal pain and discomfort.

Study	Population	Diagnostic criteria	Conclusion	Details
**Abdominal pain and discomfort**				
Lu 2000	IBS	Manning criteria	No change in severity of abdominal pain after 2 weeks of mebeverine treatment	Severity of mild, moderate and severe abdominal pain was reported in 63%, 37% and 0% patients at baseline and 67%, 30% and 2% patients after 2 weeks of treatment (not significant).
Van Outryve 1995	IBS	Kruis criteria	Abdominal pain reduced after 6 weeks of mebeverine treatment	Abdominal pain was reported in all patients at baseline and in more than 50% of patients after treatment but with lower intensity.
Schaffstein 1990	IBS without diarrhoea	Kruis criteria	Frequency and intensity of abdominal pain reduced after 4 weeks of mebeverine treatment	Mean pain frequency per day reduced from 2.49 at baseline to 1.02 at week 4. Pain intensity scores after treatment decreased by 0.58, 0.65 and 0.96 from baseline for upper, lower and diffuse pain, respectively. Mean duration of pain per episode and per day reduced from 28.61 and 85.39 min at baseline to 12.01 and 28.71 min at week 4.
Jones 1999	IBS without constipation	Rome I	Pain and discomfort reduced after 12 weeks of mebeverine treatment	Adequate relief of IBS pain and discomfort was reported in 39%, 43% and 48% of patients after 4, 8 and 12 weeks of treatment, respectively. Median proportion of pain and discomfort-free days increased from 0 at baseline to 30 after 12 weeks of treatment.

Gilbody 2000	IBS	Rome I	Abdominal pain reduced after 8 weeks of mebeverine treatment	Responder rates of patients with at least a 50% reduction in global mean VAS using for abdominal pain assessment were 71% and 70% after 8 weeks of treatment in mebeverine 135 mg 3 times daily and 200 mg twice daily, respectively.
Rahman 2014	IBS	Rome II	Abdominal pain significantly reduced after 6 weeks of mebeverine treatment	After 6 weeks of mebeverine treatment, 50% of patients had improvement in abdominal pain score ($p < 0.02$)
Sahib 2013	IBS	Rome III	Pain severity and frequency reduced after 8 weeks of mebeverine treatment	Mild, moderate and severe pain severity was reduced from 25%, 45% and 30% at baseline to 5%, 20% and 5% after 8 weeks of treatment, respectively.<br>Mild, moderate and severe pain frequency was reduced from 20%, 40% and 40% at baseline to 10%, 15% and 5% after 8 weeks of treatment, respectively.
Lee 2011	IBS with diarrhoea	Rome III	Abdominal pain/discomfort severity significantly reduced after 4 weeks of mebeverine treatment	Responder rates of patient-reported relief of abdominal pain/discomfort were 8%, 19%, 28% and 35% after 1, 2, 3 and 4 weeks of treatment, respectively. The proportion of weekly responders for adequate relief of abdominal pain/discomfort significantly increased during the study ($p < 0.001$).<br>Severity score of abdominal pain/discomfort reduced from 1.9 at baseline to 1 at week 4 ($p < 0.001$).
Guslandi 2011	IBS without constipation	Rome III	Abdominal discomfort reduced after 6 weeks of mebeverine treatment	71% of patients reported disappearance or improvement of initial abdominal discomfort after treatment.
Hou 2014	IBS	Rome III	Abdominal pain/discomfort severity reduced after 4 and 8 weeks of mebeverine treatment	Mean (SD) abdominal pain/discomfort score reduced from baseline by: 0.9 (0.73) at week 4 and 1.3 (0.80) at week 8 (Poland); 0.9 (0.77) at week 4 and 1.4 (0.78) at week 8 (Egypt); 1.4 (1.02) at week 4 and 1.9 (1.08) at week 8 (Mexico).
Chakraborty 2019	IBS with diarrhoea	Rome IV	Abdominal pain severity significantly reduced after 4 and 8 weeks of mebeverine treatment	Mean (SD) abdominal pain severity score reduced from 1.8 (0.69) at baseline to 1.4 (0.59) at week 4 and 1.3 (0.57) at week 8 ($p<0.001$).

Hatami 2020	IBS	Rome IV	Pain and severity reduced after 4 weeks of mebeverine treatment	Mean pain severity and frequency scores were reduced from baseline by 18.25 and 16.5 at week 4, respectively ($p<0.05$).
Mokhtare 2018	IBS with diarrhoea	Rome IV	Abdominal pain reduced after 4 weeks of mebeverine treatment	After 4 weeks of mebeverine treatment improving rate of abdominal pain was 62%, and the mean score of abdominal pain reduced from 5 at baseline to 1 at week 4 ($p=0.02$).
Baume 1972	IBS	Truelove and Reynell diagnostic criteria for IBS	Pain reduced after 2 weeks of mebeverine treatment	44% of patients judged the mebeverine to be superior to placebo, and this benefit applied either to a diminution in pain or to improvement in bowel function or to both of these.
Prout 1983	IBS	NR	Abdominal pain severity significantly reduced after 2 months of mebeverine treatment	Severity of abdominal pain score was significantly lower in mebeverine groups (1.675 and 1.652 for mebeverine low and high dose respectively) than in the placebo group (1.947; $p < 0.05$).
Kruis 1986	IBS	Own criteria	Abdominal pain reduced only in a few patients after 16 weeks of mebeverine treatment	23% of patients reported disappearance or improvement of initial abdominal pain after treatment.
Inauen 1994	IBS	NR	Abdominal pain reduced after 3 weeks of mebeverine treatment	After 3 weeks of mebeverine treatment, abdominal pain had disappeared or improved in 89% of patients.
Tudor 1986	IBS	NR	Pain and discomfort significantly reduced after 4 weeks of mebeverine treatment	There was a significant reduction in symptoms of pain and discomfort after mebeverine treatment ($p < 0.02$).
Capurso 1984	IBS	NR	Pain severity significantly reduced after 2 weeks of mebeverine treatment	Severity of pain was significantly lower in the mebeverine group than in the placebo group ($p < 0.001$).

NR – not reported.

Table S7. Summary of the results of mebeverine studies with respect to the symptoms associated with abdominal distension.

Study	Population	Diagnostic criteria	Conclusion	Details
**Abdominal distension and cramps**				
Sahib 2013	IBS	Rome III	Abdominal distension reduced after 8 weeks of mebeverine treatment	Mild, moderate and severe abdominal distension reduced from 25%, 45% and 30% at baseline to 15%, 10% and 0% after 8 weeks of treatment, respectively.
Prout 1983	IBS	NR	Abdominal distension significantly reduced after 2 months of mebeverine treatment	Severity of distension score was significantly lower in mebeverine groups (1.718 and 1.692 for mebeverine low and high dose respectively) than in the placebo group (1.839; $p < 0.05$).
Inauen 1994	IBS	NR	Abdominal distension reduced after 3 weeks of mebeverine treatment	After 3 weeks of mebeverine treatment, abdominal distension had disappeared or improved in 83% of patients.

NR – not reported.

Table S8. Summary of the results of mebeverine studies with respect to the symptoms associated with abnormal bowel habits and bloating.

Study	Population	Diagnostic criteria	Conclusion	Details
**Abnormal or irregular bowel habits**				
Lee 2011	IBS with diarrhoea	Rome III	Abnormal bowel habits significantly reduced after 4 weeks of mebeverine treatment	Responder rates of patient-reported relief of abnormal bowel habits were 9%, 17%, 28% and 34% after 1, 2, 3 and 4 weeks of treatment, respectively. The proportion of weekly responders for adequate relief of abnormal bowel habits significantly increased during the study ($p < 0.001$).
Baume 1972	IBS	Truelove and Reynell diagnostic criteria for IBS	Abnormal bowel habits reduced after 2 weeks of mebeverine treatment	44% of patients judged the mebeverine to be superior to placebo, and the benefit which patients noted applied either to a diminution in pain or to improvement in bowel function or to both of these.
Prout 1983	IBS	NR	Pain on moving bowels and wind significantly reduced	Severity of pain on moving bowels score was significantly lower in mebeverine groups (1.188 and 1.248 for mebeverine low and high dose

			after 2 months of mebeverine treatment	respectively) than in the placebo group (1.374; p < 0.05). Severity of wind score was significantly lower in mebeverine groups (1.957 and 1.979 for mebeverine low and high dose respectively) than in the placebo group (2.193; p < 0.05).
Kruis 1986	IBS	Own criteria	Irregular bowel habits reduced only in a few patients after 16 weeks of mebeverine treatment	13% of patients reported disappearance or improvement of initial irregular bowel habits after treatment.
Tudor 1986	IBS	NR	Normal bowel habits improved after 4 weeks of mebeverine treatment	About 60% of patients reported normal bowel habits over the whole trial, and this figure rose on mebeverine treatment
		**Bloating**		
Lu 2000	IBS	Manning criteria	No statistically significant change but numerical reduced after 2 weeks of mebeverine treatment	Bloating severity was reported in 46% of patients at baseline and 27% of patients at week 2 (not significant).
Van Outryve 1995	IBS	Kruis criteria	Reduced after 6 weeks of mebeverine treatment	Bloating, flatulence and constipation all disappeared in more than 62% of patients after treatment.
Chang 2011	IBS	Rome II	Significantly reduced after 8 weeks of mebeverine treatment	Abdominal bloating assessed on the VAS scale reduced from the median (IQR) 4.7 (6.6) at baseline to 1.3 (4.6) at week 8 (p < 0.001).
Guslandi 2011	IBS without constipation	Rome III	Reduced after 6 weeks of mebeverine treatment	61% of patients reported disappearance or improvement of initial bloating after treatment.
Mokhtare 2018	IBS with diarrhoea	Rome IV	Reduced after 4 weeks of mebeverine treatment	The mean score of bloating reduced from 5 at baseline to 1 at week 4 (p=0.033).

NR – not reported.

Table S9. Summary of the results of mebeverine studies with respect to the symptoms associated with constipation and diarrhea.

Study	Population	Diagnostic criteria	Conclusion	Details
**Constipation**				
Van Outryve 1995	IBS	Kruis criteria	Reduced after 6 weeks of mebeverine treatment	Bloating, flatulence and constipation all disappeared in more than 62% of patients after treatment.
Inauen 1994	IBS	NR	Reduced after 3 weeks of mebeverine treatment	After 3 weeks of mebeverine treatment, obstipation had disappeared or improved in 79% of patients.
Capurso 1984	IBS	NR	Significantly reduced after 2 weeks of mebeverine treatment	Severity of constipation/diarrhoea was significantly lower in the mebeverine group than in the placebo group ($p < 0.001$).
Diarrhoea				
Guslandi 2011	IBS without constipation	Rome III	Reduced after 6 weeks of mebeverine treatment	64% of patients reported disappearance or improvement of initial diarrhoea after treatment.
Capurso 1984	IBS	NR	Significantly reduced after 2 weeks of mebeverine treatment	Severity of constipation/diarrhoea was significantly lower in the mebeverine group than in the placebo group ($p < 0.001$).
Mokhtare 2018	IBS with diarrhoea	Rome IV	Reduced after 4 weeks of mebeverine treatment	After 4 weeks of mebeverine treatment improving rate of diarrhoea was 56%.
**Urgency**				
Jones 1999	IBS without constipation	Rome I	Reduced after 12 weeks of mebeverine treatment	The mean proportion of days with urgency was reduced from 68% at baseline to 46% after 12 weeks of treatment.
Sahib 2013	IBS	Rome III	Reduced after 8 weeks of mebeverine treatment	Mild, moderate and severe urgency reduced from 20%, 35% and 45% at baseline to 15%, 15% and 5% after 8 weeks of treatment, respectively.
Lee 2011	IBS with diarrhoea	Rome III	Significantly reduced after 4 weeks of mebeverine treatment	Severity score of urgency reduced from 1 at baseline to 0.6 at week 4 ($p < 0.001$).

NR – not reported.

Table S10. Summary of the results of mebeverine studies with respect to the symptoms associated with stool frequency and consistency.

Study	Population	Diagnostic criteria	Conclusion	Details
**Stool frequency**				
Lu 2000	IBS	Manning criteria	Significantly improved after 2 weeks of mebeverine treatment	Frequency of defecation reduced from mean (SD) 2.7 (1.1) times per day at baseline to 2.1 (1.0) times per day at week 2 ($p < 0.05$).
Jones 1999	IBS without constipation	Rome I	Improved after 12 weeks of mebeverine treatment	The number of stools per day reduced from 2.76 at baseline to 2.27 after 12 weeks of treatment.
Chang 2011	IBS	Rome II	Significantly improved after 12 weeks of mebeverine treatment	Stool frequency assessed on the VAS scale reduced from the median (IQR) 4.8 (5.3) at baseline to 1.7 (3.9) at week 8 ($p < 0.001$).
Rahman 2014	IBS	Rome II	Significantly improved after 6 weeks of mebeverine treatment	Improvement in respect to altered stool frequency >3 times per day and altered stool frequency <1 time per day after 6 weeks of treatment was statistically significant in regard to baseline ($p < 0.01$ and $p < 0.05$, respectively).
Sahib 2013	IBS	Rome III	Improved after 8 weeks of mebeverine treatment	Mild, moderate and severe stool frequency reduced from 35%, 40% and 25% at baseline to 5%, 15% and 0% after 8 weeks of treatment, respectively.
Lee 2011	IBS with diarrhoea	Rome III	Significantly improved after 4 weeks of mebeverine treatment	Stool frequency reduced from 2.5 per day at baseline to 2.1 per day at week 4 ($p < 0.001$).
Chakraborty 2019	IBS with diarrhoea	Rome IV	Significantly improved after 4 and 8 weeks of mebeverine treatment	The mean (SD) number of daily bowel movements changed from 5.6 (2.06) at baseline to 4.3 (2.18) at week 4 and 3.9 (2.34) at week 8 ($p<0.001$).
Mokhtare 2018	IBS with diarrhoea	Rome IV	No statistically significant change after 4 weeks of mebeverine treatment	The mean score of stool frequency reduced from 3 at baseline to 2 at week 4 ($p=0.086$).
**Stool consistency**				
Lu 2000	IBS	Manning criteria	Significantly improved after 2 weeks of mebeverine treatment	Stool consistency hard and watery was reported respectively in 2% and 20% patients at baseline and 11% and 2% at week 2 ($p < 0.01$).

Jones 1999	IBS without constipation	Rome I	Improved after 12 weeks of mebeverine treatment	Stool consistency score improved from 3.46 at baseline to 3.15 after 12 weeks of treatment (scores from 1 – very hard to 4 – loose).
Rahman 2014	IBS	Rome II	Significantly improved after 6 weeks of mebeverine treatment	Improvement in respect to soft stool and hard stool after 6 weeks of treatment was statistically significant in regard to baseline ($p < 0.01$).
Sahib 2013	IBS	Rome III	Improved after 8 weeks of mebeverine treatment	Mild, moderate and severe stool consistency reduced from 30%, 35% and 35% at baseline to 20%, 10% and 5% after 8 weeks of treatment, respectively.
Lee 2011	IBS with diarrhoea	Rome III	Significantly improved after 4 weeks of mebeverine treatment	Stool form evaluated using the Bristol stool form scale improved from score 5.5 at baseline to 4.6 at week 4 (scores from 1 – separate hard lumps to 7 – watery; $p < 0.001$).

Table S11. Summary of the results of mebeverine studies with respect to the symptoms associated with nausea, anxiety and depression.

Study	Population	Diagnostic criteria	Conclusion	Details
			**Nausea**	
Prout 1983	IBS	NR	Significantly reduced after 2 months of mebeverine treatment	Severity of the nausea score was significantly lower in the mebeverine low dose group (1.170) than in the placebo group (1.311; $p < 0.05$).
			**Anxiety**	
MIBS trial	IBS	Rome III	Reduced after 6 and 12 weeks of mebeverine treatment	The total score of HADS for anxiety reduced from a mean 9.23 at baseline to 8.7 at week 6 and to 8.2 at week 12.
Prout 1983	IBS	NR	Significantly reduced after 2 months of mebeverine treatment	Severity of anxiety score was significantly lower in the mebeverine high dose group (1.578) than in the placebo group (1.704; $p < 0.05$).
			**Depression**	
MIBS trial	IBS	Rome III	Reduced after 6 and 12 weeks of mebeverine treatment	The score of HADS for depression was reported as normal by 85% and 78% of patients after 6 and 12 weeks of treatment, respectively.

NR – not reported.

Table S12. Summary of the safety results.

Study name	Safety results
Lu 2000	All patients completed the study without any severe adverse events.
Van Outryve 1995	20% of patients during the mebeverine plain treatment period, and 13% of patients during the mebeverine SR treatment period experienced drug-related adverse events, which were mild to moderate in severity. The overall compliance was 99% for mebeverine SR; for the mebeverine plain, it was 99% after 3 weeks and 98% after 6 weeks (no significant difference).
Schaffstein 1990	At least one side effect was reported by 2 and 8 patients in the trimebutine and mebeverine arm, respectively.
Jones 1999	40% of patients during the alosetron treatment period, and 27% of patients during the mebeverine treatment period experienced adverse events. Serious adverse events occurred in 3% of patients both in the alosetron and mebeverine groups. Serious adverse events were reported to be unrelated to the study medication.
Gilbody 2000	The frequency of adverse events, which included symptoms associated with IBS, was comparable in both treatment arms, and no safety concerns were identified. There was a high level of reporting of symptoms, particularly of a digestive nature, the commonest of which were abdominal pain and diarrhoea. During treatment, 62% and 59% of patients experienced at least one adverse event in mebeverine 135 mg and 200 mg arm, respectively. There were 6 serious adverse events, and 5 of them were reported to be unrelated to the study medication.
Chang 2011	The most often reported side effect in the mebeverine group was dry mouth. In the otilonium bromide arm, 14% of patients had nausea, while none were in the mebeverine group ($p = 0.006$).
Rahman 2014	No worsening of symptoms and no side effects in both treatment arms was observed in any patient during the trial.
Sahib 2013	Not reported
MIBS trial 2013	Not reported
Lee 2011	All of the adverse events were comparable between the two groups and described as mild or moderate. No serious adverse events such as severe constipation and ischemic colitis were reported in the ramosetron and mebeverine groups.
Guslandi 2011	Not reported
Hou 2014	There were 2 AE considered possibly drug-related (moderate severity abdominal distension and mild severity somnolence), both of which were non-serious and occurred in the mebeverine group.
Chakraborty 2019	There was one probable AE in the mebeverine group. One patient developed a heartburn sensation within a few days after initiation of mebeverine treatment.
Hatami 2020	The frequency of AE was higher in the cumin sofouf group compared to the mebeverine group. There were 3 AE reported among patients treated with mebeverine and 12 in the cumin sofouf group. The only symptoms in the mebeverine group were gastrointestinal upset and headache.
Mokhtare 2018	Mild, tolerable and transient AE were reported in 2 patients (dyspepsia and constipation); all of them occurred in the mebeverine group.

Connell 1965	Side effects during the course of the trial were reported in 5 patients, including 3 in the mebeverine group and 2 in the placebo group. AE in mebeverine group: depression and headache, depression and dizziness, headaches. AE in the placebo group: wind accumulating in epigastrium, headaches (occipital), loss of concentration and dizziness.
Baume 1972	Side effects were rare and were equally shared between mebeverine and placebo groups
Prout 1983	The trial medication was well tolerated; no differences between any dose of mebeverine and placebo were reported regarding adverse effects.
Kruis 1986	Clinically relevant side effects were not observed during the study period. The only symptoms were nausea, abdominal pain, dryness of the mouth, general malaise and constipation.
Inauen 1994	Both mebeverine formulations were safe and well-tolerated, and no serious adverse drug events were reported. The investigator's global impression of tolerability after 1 and 3 weeks of treatment did not significantly differ between the groups. The investigator's judgement on compliance was good or excellent in 96% of the patients
Tudor 1986	There were 8 reports of adverse events, including 6 in the mebeverine arm. Those reported on mebeverine were nausea, headache, tiredness and dizziness.
Capurso 1984	Cephalea, dryness of the mouth and tachycardia were observed in 3 mebeverine treated patients. No adverse events were observed in the octilonium bromide treated patients.

Table S13. Comparison of different IBS diagnostic criteria.

IBS criteria	Year	Symptoms, signs and laboratory results
The Manning criteria	1978	– Onset of pain linked to more frequent bowel<br>– Looser stools associated with onset of pain<br>– Pain relieved by passage of stool<br>– Noticeable abdominal bloating<br>– Sensation of incomplete evacuation > 25% of the time<br>– Diarrhea with mucus > 25% of the time
The Kruis criteria	1984	– Symptoms of abdominal pain, flatulence, or bowel irregularity<br>– Symptom duration > 2 years<br>– Pain described as burning, cutting, very strong, terrible, feeling of pressure, dull, boring, or 'not so bad'<br>– Alternating constipation and diarrhea
Rome I	1989	Abdominal pain or discomfort relieved by defecation, or associated with a change in stool frequency or consistency, and two more of the following on at least 25% occasions or days (duration of symptoms at least 3 months):<br>– Altered stool frequency<br>– Altered stool form<br>– Altered stool passage<br>– Passage of mucus<br>– Bloating or distention
Rome II	1999	Patient must have at least 12 weeks of abdominal discomfort or pain, which need not be consecutive, in the preceding 12 months with at least 2 of following 3 features:<br>– Relieved with defecation<br>– Onset associated with a change in stool frequency<br>– Onset associated with a change in stool form (appearance)
Rome III	2006	Recurrent abdominal pain or discomfort, 3 days per month in the last 3 months (12 weeks), associated with ≥2 of the criteria below. The criteria are fulfilled with symptoms onset 6 months prior to diagnosis.<br>– Improvement with defecation<br>– Onset associated with a change in stool frequency<br>– Onset associated with a change in stool form (appearance)
Rome IV	2016	Recurrent abdominal pain on average 1 day per week in the last 3 months (12 weeks), associated with ≥2 of the criteria below. The criteria are fulfilled with symptoms onset 6 months prior to diagnosis.<br>– Related to defecation (either increasing or improving pain)<br>– Associated with a change in stool frequency<br>– Associated with a change in stool form (appearance)

Table S14. Prevalence of IBS in patients diagnosed by different criteria.

Study name	Population	Number of pts	Diagnostic criteria				
			Manning	Rome I	Rome II	Rome III	Rome IV
Ghoshal 2013	Patients with chronic lower gastrointestinal symptoms	1618	91%	68%	40%	53%	x
Sperber 2007	Representative sample of Israeli adults	981	x	x	3%	11%	x
Park 2010	General Korean population	1009	x	x	8%	9%	x
Bai 2016	Outpatients in the gastrointestinal department of a tertiary hospital	1376	x	x	x	12%	6%
Patcharatrakul 2017	Patients with any gastrointestinal symptoms at general medicine clinic of a tertiary hospital	438	x	x	x	24%	17%
Vork 2018	IBS patients fulfilled Rome III criteria from the secondary/tertiary care outpatient department	404	x	x	x	100%	87%
Palsson 2020	The general population of 3 countries (US, UK and Canada) completed a secure online survey.	5931	x	x	x	11%	6%
